# Use of artificial neural network to predict esophageal varices in patients with HBV related cirrhosis

**Published:** 2011-07-01

**Authors:** Wan-dong Hong, Yi-feng Ji, Dang Wang, Tan-zhou Chen, Qi-huai Zhu

**Affiliations:** 1 Department of Gastroenterology and Hepatology, the First Affiliated Hospital of Wenzhou Medical College, Wenzhou, China; 2 Department of Internal Medicine, the People's Hospital of Wencheng County, Wencheng, China

**Keywords:** Predictor, Esophageal varices, Neural network

## Abstract

**Background:**

Prediction of esophageal varices in cirrhotic patients by noninvasive methods is still unsatisfactory.

**Objectives:**

To evaluate the accuracy of an artificial neural network (ANN) in predicting varices in patients with HBV related cirrhosis.

**Patients and Methods:**

An ANN was constructed with data taken from 197 patients with HBV related cirrhosis. The candidates for input nodes of the ANN were assessed by univariate analysis and sensitivity analysis. Five-fold cross validation was performed to avoid over-fitting.

**Results:**

14 variables were reduced by univariate and sensitivity analysis, and an ANN was developed with three variables (platelet count, spleen width and portal vein diameter). With a cutoff value of 0.5. The ANN model has a sensitivity of 96.5%, specificity of 60.4%, positive predictive value of 86.9%, negative predictive value of 86.5% and a diagnostic accuracy of 86.8% for the prediction of varices.

**Conclusions:**

An ANN may be useful for predicting presence of esophageal varices in patients with HBV related cirrhosis.

## 1. Background

Despite significant improvements in the early diagnosis and treatment of esophageal variceal bleeding, the mortality rate of the first episode of variceal bleeding still remains high [1]. Early identification of patients with esophageal varices (EV) may be helpful for primary prophylaxis. Though the upper gastrointestinal endoscopy remains the gold standard for the diagnosis of gastroesophageal varices, non invasive diagnostic means are desired to reduce the frequency of endoscopic examinations and related costs. A number of studies have addressed the issue and identified a series of predictors such as platelet count [2], spleen width, portal vein diameter [1][2][3], and platelet count/bipolar spleen diameter ratio [4]. However, different studies performed in cirrhotic patients have yielded different results[5]. Such predictive factors may be expected to vary in different populations because of differences in the etiologies of liver cirrhosis and severity of liver disease. It has been known that cirrhotic patients in China, Southeast Asia usually have a higher proportion with HBV etiology[1]. A recent study has demonstrated that an artificial neural network (ANN) analysis is potentially more successful than the conventional statistical techniques in predicting clinical outcomes when the relationship between variables that determine the prognosis is complex, multidimensional and non-linear [6].

## 2. objectives

The aim of this study was to evaluate the accuracy of an ANN in predicting EV in patients with HBV related cirrhosis.

## 3. Patients and Methods

197 patients with HBV-related cirrhosis were enrolled in this study between July 2005 and August 2008. Exclusion criteria have been previously described in detail [1]. Age, gender and biochemical parameters were recorded. Spleen width and portal vein diameter were measured by ultrasonography [7]. The presence and degree of ascites and encephalopathy was assessed according to Child-Pugh criteria [8]. All gastrointestinal endoscopies were performed by several senior endoscopists (WDH, QHZ and others). All endoscopic results were re-evaluated by two endoscopists (WDH and QHZ) who were unaware of the patients' clinical and ultrasonographic results. Any disagreements on the size of EV were resolved by discussion. Kappa statistic was used to measure the level of inter-observer agreement for the size of EV was 0.80. The size of varices was subdivided into two classes: "small" and "large" [9]. Diuretics therapy was not commenced before endoscopy and ultrasonography. The study protocol was approved by the Ethic Committee of the First Affiliated Hospital of Wenzhou Medical College. A Shapiro Wilk test was used to evaluate if the continuous data had normal distribution. Normally distributed variables were presented as mean ± SD and compared with Student's t test. Non normally distributed variables were presented as median and interquartile range and compared by Mann-Whitney U test. Categorical values were compared by x² test. Variables found to be significantly correlated to the presence of EV were selected as candidates for inputs of the ultimate ANN model. Sensitivity analysis (also known as independent variable importance analysis) was also performed by SPSS 16.0 software to determine the optimum variables which would be used to construct the ultimate ANN model [10]. When performing sensitivity analysis, data were randomly divided into a training sample (n=110, 56%), testing sample (n = 30, 15%) and holdout sample (n = 57, 29%).

An exploratory, three-layered, multiplayer perceptron ANN model, with back propagation algorithm was constructed for sensitivity analysis. Sigmoid transfer functions were used in the hidden and output layers. Gradient descent was used to estimate the synaptic weights. The learning rate was 0.1 and the momentum was 0.1 [11]. According to the results of univariate and sensitivity analyses, for all studied 197 patients, an ultimate, three-layered, feed-forward ANN model with three hidden nodes, with back propagation algorithm was constructed by JMP 6.0 software.

Generally speaking, neural network models are highly over-parameterized, so that models that seem to offer the best fit of the (training) data are over-fit for other data. To prevent over-fitting in large data sets, it is suggested that the data should be partitioned into a "training group" used for fitting the model and a "testing group" for comparing models that have been fitted to the training data [12]. However, for a small dataset a k-fold cross-validation model is more suitable and reliable to prevent over-training [12][13]. So, in the present study, we performed five-fold cross-validation in all 197 patients. The ANN model was trained with maximum iterations of 500 and 10 tours. The over-fit penalty was set to 0.001; the convergence criterion was set to 0.00001. The output of ANN model was transformed to range (0-1). Presence of EV was predicted if the output was greater than or equal to 0.5 [12]. The sensitivity, specificity, negative predictive value, positive predictive value and diagnostic accuracy of the ANN model were reported. Differences were considered to be statistically significant if the two-tailed p value was less than 0.05.

## 4. Results

Of 197 studied patients, 130 (66%) were male; the patients had a mean ± SD age of 53.5 ± 12 years. The prevalence of EV was 73.1%. The majority of the patients had Child-Pugh class A (39.1%) and B (41.1%). Ascites was found in 30.5% of the patients by ultrasonography and clinical examination.

4.1. Artificial neural network analysis

**Figure 1:**
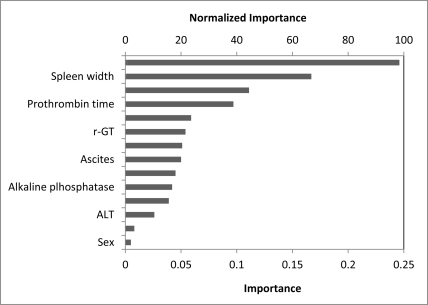
Sensitivity analysis of input variables. The value shown for each input variable is a measure of its relative importance.

**Figure 2:**
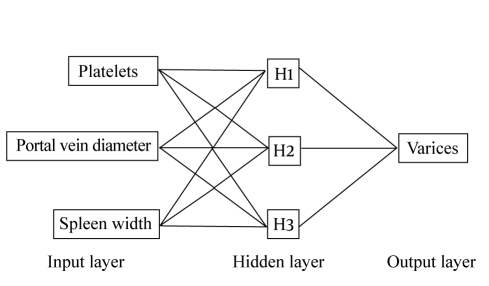
Schematic diagram of the ANN model developed to predict the presence of EV

Fourteen variables considered relevant to the presence of EV were tested using univariate analysis (Table 1). Four variables (platelet count, ascites, spleen width and portal vein diameter) were significantly associated with the presence of EV, of which platelet count, spleen width and portal vein diameter were also noted as the most important predictors of EV by sensitivity analysis (Figure 1) (The exploratory ANN model constructed by SPSS 16.0 for sensitivity analysis is not shown). Therefore, an ultimate three-layer 3-3-1 feed-forward back-propagation ANN model, which was consisted of platelet count, spleen width and portal vein diameter, was developed and trained by JMP 6.0 software in 197 patients (Figure 2). Sensitivity, specificity, positive predictive value, negative predictive value and diagnostic accuracy of ANN in comparison with endoscopy examination in the diagnosis of EV in patients with HBV related cirrhosis are shown in Table 2.

**Table 1:** Univariate analysis of predictors of esophageal varices in 197 patients

**Variables**	**Patients without EV** (n = 53)	**Patients with EV** (n = 144)	***P* value**
**Age**, y, Mean ± SD	55.3 ± 13.3	52.8 ± 11.5	0.20 b
**Male**, %	66.0	65.9	0.99 a
**Total bilirubin**, μmol/L	23 (15–40)	29.5 (21–43.5)	0.21 c
**Albumin**, g/L	34 (26–38.7)	31 (27.5–35.7)	0.23 c
**ALT**, U/L	37 (31–61)	43.5 (29.5–66)	0.69 c
**AST**, U/L	58 (35–85)	64 (41–84)	0.38 c
**Alkaline phosphatase**, U/L	99 (70–123)	101 (72–131)	0.63 c
**γ-GT**, U/L	55 (33–93)	60.5 (36.5–112.5)	0.43 c
**Prothrombin time**, s	16.5 (15.3–18.9)	17.5 (15.6–19.8)	0.19 c
**Prothrombin activity**, %	64 (54–76)	60.5 (50–70.5)	0.15 c
**Platelets**, 10^9^/L	75 (56–109)	49 (32–66)	< 0.001 c
**Ascites**, No.			0.03 a
**None**	44	93	
**Non-tense**	6	25	
**Tense**	3	26	
**Portal vein diameter**, mm	11 (10–12)	13 (12–14)	< 0.001 c
**Spleen width**, mm	40 (38–44)	51 (44–60)	< 0.001 c

^a^ Student's t-test

^b^ X(2) test

^c^ Mann-Whitney U-test

**Table 2:** Artificial neural network performance in predicting the presence of esophageal varices

	**EV b**	**No EV b**	**Total**
**ANN result a**			
** Positive **	135	15	150
** Negative**	9	38	47
**Total**	144	53	197

^a^ ANN: Artificial neural network; Sensitivity: 93.75%; Specificity: 71.70%; Positive predictive value: 90.00%; Negative predictive value: 80.85%; Diagnostic accuracy: 87.82%

^b^ EV: Esophageal Varices

## 5. Discussion

Data on the relationship between thrombocytopenia and the risk of EV is somewhat conflicting. Several studies suggest that platelet count may predict the presence of EV in patients with cirrhosis [2][14]. However, recent data obtained by logistic regression analysis do not support any correlation between thrombocytopenia and varices [1][5]. Our data by ANN analysis showed that thrombocytopenia was one of the independent risk factors for the presence of EV. This may in part be attributed to the fact that ANN analysis is potentially more successful than conventional statistical techniques in predicting clinical outcomes when the relationship between predictors and outcomes is complex and nonlinear. This is evidenced by the fact that thrombocytopenia in cirrhotic patients is not only attributed to hypersplenism due to portal hypertension but also resulted from decreased thrombopoeitin and interleukin-11 [1]. Splenomegaly is a common finding in cirrhotic patients with portal hypertension. Lamb et al. found that there was a good correlation between in vivo ultrasound assessment of splenic width and true splenic volume [7]. The present study showed that spleen width was an independent predictor for the presence of EV, which is also consistent with our previous observations [1][3]. Contrary to what was suggested in previous reports, no significant correlation between splenomegaly and presence of EV was found in other studies [2][15]. These differences may be due to the variations among studies regarding the etiology and the stage of liver cirrhosis they studied. EV is the direct consequence of spontaneous formation of collateral vessels between portal vein and esophageal veins via left gastric or short gastric veins. Therefore, the presence or absence of EV may reflect the severity of portal hypertension. The results of the present study indicate that the portal vein diameter could be a valuable predictor of EV in patients with HBV related cirrhosis. This finding was consistent with previous reports [1][3]. As shown in Table 2, the ANN model, which was consisted of platelet count, spleen width and portal vein diameter, achieved a positive predictive value of 90.0% and a negative predictive value of 80.85%. This means that if the ANN value is more than or equal to 0.5, there is a probability of 90.0% for presence of EV. And, if ANN value is less than 0.5, there is a probability of 80.85% for absence of EV. Overall, 87.82% of patients were correctly classified.

Our study has several limitations. Data were collected retrospectively, which might produce a population bias. In addition, the sample size was small and the grade of esophageal varices in studied patients was not compared. At last, although we preformed 5-fold cross validation and filtered out irrelevant input variables by univariate and sensitivity analysis to avoid over-fitting, testing the performance of ANN model with an independent sample set would be necessary in the future. In conclusion, an ANN model, which was consisted of platelet count, spleen width and portal vein diameter, may be useful for predicting presence of EV in patients with HBV related cirrhosis.
